# Sorghum as a monocot model for drought research

**DOI:** 10.3389/fpls.2025.1665967

**Published:** 2025-09-24

**Authors:** Juan B. Fontanet-Manzaneque, Daniela M. Hernández, Andrea Giordano, Ana I. Caño-Delgado

**Affiliations:** ^1^ Department of Molecular Genetics, Centre for Research in Agricultural Genomics (CRAG) CSIC-IRTA-UAB-UB, Barcelona, Spain; ^2^ PLANeT Biotech, Parc de Recerca UAB, Barcelona, Spain

**Keywords:** Sorghum, monocot model, drought, omics, transformation

## Abstract

Climate change is intensifying drought events, posing a major threat to global food security. *Sorghum bicolor* (L.) Moench (Sorghum), a C4 monocot grass, is emerging as a valuable model for drought research due to its natural tolerance to water limitation and adaptability to semi-arid and arid environments. Sorghum cultivation requires significantly less water than major cereals such as rice, maize, and wheat, making it an attractive crop for sustaining agricultural productivity under water-limiting conditions. In fact, Sorghum uses up to 34% less water than rice in rainfed systems and up to 50% less under irrigation, with rice-to-Sorghum substitution potentially reducing water demand by 33%. Its lower water requirements, along with the compact growth of commonly used accessions such as TX430 and BTx623, make Sorghum a practical system for experimentation, particularly in genome editing studies. Maize, which shares close genetic similarity and also belongs to the Panicoideae subfamily, could particularly benefit from Sorghum-based insights. Sorghum also overcomes key limitations of model species such as *Arabidopsis thaliana*, offering greater relevance to monocot crops. Additionally, advances in metabolomics, transcriptomics, proteomics, phenomics, population genomics and pangenomics are expanding our understanding of the molecular and physiological mechanisms underlying Sorghum’s drought resilience. Despite these advantages, challenges remain in transformation efficiency and the availability of genomic tools. This review highlights Sorghum’s drought tolerance mechanisms, available omics and genetic tools, described drought-related genes and regulatory networks, and the limitations and progress in gene manipulation for climate-resilient crop development. Sorghum uniquely combines the advantages of a staple crop and a model organism, making it a powerful next-generation system for climate-resilient agriculture.

## 1 Introduction

Climate change has intensified the frequency of extreme events, such as heatwaves and droughts, since the 1950s, exposing millions to food and water insecurity, particularly in Africa, Asia, and Central and South America (IPCC, 2023). Heat and drought are the primary contributors to crop yield reduction under climate change (Rezaei et al., 2023). However, drought alone represents the greatest limitation to food production, causing more annual crop yield loss than all pathogens combined, with an estimated $30 billion in losses over the past decade (Gupta et al., 2020). Cereal crops are especially vulnerable: a 40% reduction in water availability leads to a 40% decrease in maize yield and a 20% decrease in wheat yield, while a 50% reduction in water results in a 60% decline in rice yield and a 30% decline in Sorghum yield (Daryanto et al., 2017). Moreover, even short periods of drought can significantly impact final yield outcomes, as the timing of drought within the plant cycle is critical, especially during germination, seedling establishment, floral induction, and grain development (Dietz et al., 2021; Barnabás et al., 2008).

In addition to climate change, demographic changes contribute to food security challenges. The global population is projected to increase from the current 8 billion to between 9.7 and 10.9 billion by the end of the century, raising global food demand by 35-62% by 2050 (United Nations, 2022; Van Dijk et al., 2021).

In conclusion, the combined pressures of climate change and a growing global population underscore the urgent need for crops better adapted to changing environments to meet the increasing food demand and ensure food security. However, many major cereal crops remain poorly equipped to withstand increasingly frequent and severe drought conditions. While *Arabidopsis thaliana* (Arabidopsis) has been instrumental in advancing our understanding of plant stress responses, its evolutionary distance from monocots limits its direct applicability to cereal crop improvement. To accelerate the development of climate-resilient cereals, new model systems are needed that combine experimental tractability with close genetic and physiological relevance to key crops. *Sorghum bicolor* (L.) Moench (Sorghum) emerges as a promising candidate.

Sorghum, a C4 monocot grass that diverged from maize approximately 15 million years ago, is the fifth most important cereal crop worldwide, with an annual production of around 57 million tons (Mullet et al., 2002; FAOSTAT, 2022). While serving as a staple food source for millions in Africa and Asia, Sorghum remains underutilized in more industrialized countries, where it is primarily grown for animal feed. Nonetheless, Sorghum holds considerable potential for human consumption, particularly as a gluten-free alternative. Sorghum-based food products, including bread, pasta, porridges, and parboiled rice-like products, present valuable dietary options, especially for people with celiac disease (Taylor et al., 2006).

In addition to its nutritional value, sweet Sorghum has emerged as a particularly promising bioenergy crop, particularly for bioethanol production, due to its high biomass yields on marginal lands not suitable for food or feed cultivation. Breeding efforts have enhanced traits such as sugar accumulation and secondary cell wall biosynthesis, further supporting its utility in biofuel applications (Guigou et al., 2011; Zhang et al., 2018).

**Figure 1:**
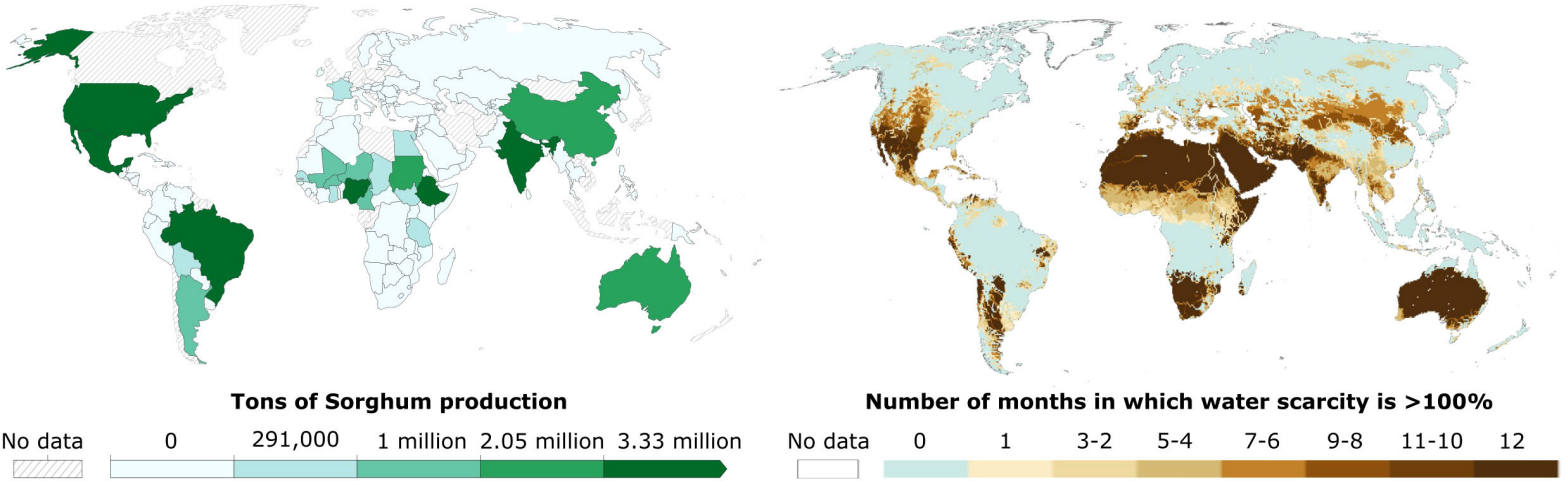
Drought is the sorghum’s most common abiotic stressor. Sorghum production is associated with water scarcity regions. In the left map there is the production of sorghum in 2023 (Food and Agriculture Organization of the United Nations (2025) – with major processing by Our World in Data) and in the right map the number of months/year with water scarcity (Adapted from: Figure Box 4.1.1 in Caretta, M.A., A. Mukherji, M. Arfanuzzaman, R.A. Betts, A. Gelfan, Y. Hirabayashi, T.K. Lissner, J. Liu, E. Lopez Gunn, R. Morgan, S. Mwanga, and S. Supratid, 2022: Water. In: Climate Change 2022: Impacts, Adaptation, and Vulnerability. Contribution of Working Group II to the Sixth Assessment Report of the Intergovernmental Panel on Climate Change [H.-O. Pörtner, D.C. Roberts, M. Tignor, E.S. Poloczanska, K. Mintenbeck, A. Alegría, M. Craig, S. Langsdorf, S. Löschke, V. Möller, A. Okem, B. Rama (eds.)]. Cambridge University Press, Cambridge, UK and New York, NY, USA, pp. 551-712, doi:10.1017/9781009325844.006).

The primary benefit of Sorghum cultivation is its drought tolerance mechanisms. Sorghum is predominantly grown in semi-arid and arid tropics of Africa and South Asia, with significant production also in China, Southeast Asia, and the Americas (Venkateswaran et al., 2019). Consequently, drought stress is considered the most common abiotic stressor that Sorghum encounters in its major production regions (**Figure 1**). Sorghum’s high protein content and minimal irrigation requirements make it an attractive alternative to water-intensive crops such as rice. For instance, replacing rice with sorghum could reduce water demand by 33% while increasing protein production by 1% (Davis et al., 2018). Moreover, sorghum demonstrates greater resilience to high temperatures compared to wheat, reinforcing its value under climate change scenarios (DeFries et al., 2023).

Beyond its agronomic and ecological advantages, sorghum possesses several features that enhance its value as a model system. It has a relatively small diploid genome, in contrast to the tetraploid durum wheat, the allohexaploid bread wheat, or maize, which—though diploid—originated from a tetraploid ancestor, increasing its genetic redundancy (Swigonova et al., 2004; Maccaferri et al., 2019; Cavalet-Giorsa et al., 2024). Sorghum also develops a primary root that is amenable to physiological studies and confocal imaging in a manner comparable to the model dicot Arabidopsis (Blasco-Escámez et al., 2017; Fontanet-Manzaneque et al., 2024b; Rico-Medina et al., 2025). Furthermore, its life cycle is shorter than that of wheat and maize and can be further reduced through embryo rescue techniques (Rizal et al., 2014).

Together, these features, combined with the availability of omics resources, advancing genetic tools, recent progress in overcoming transformation recalcitrance, and an expanding body of knowledge on drought-responsive regulatory networks, position sorghum as a next-generation model for studying drought adaptation and for driving crop improvement strategies aimed at enhancing global food security.

## 2 Sorghum drought tolerance mechanisms

**Figure 2:**
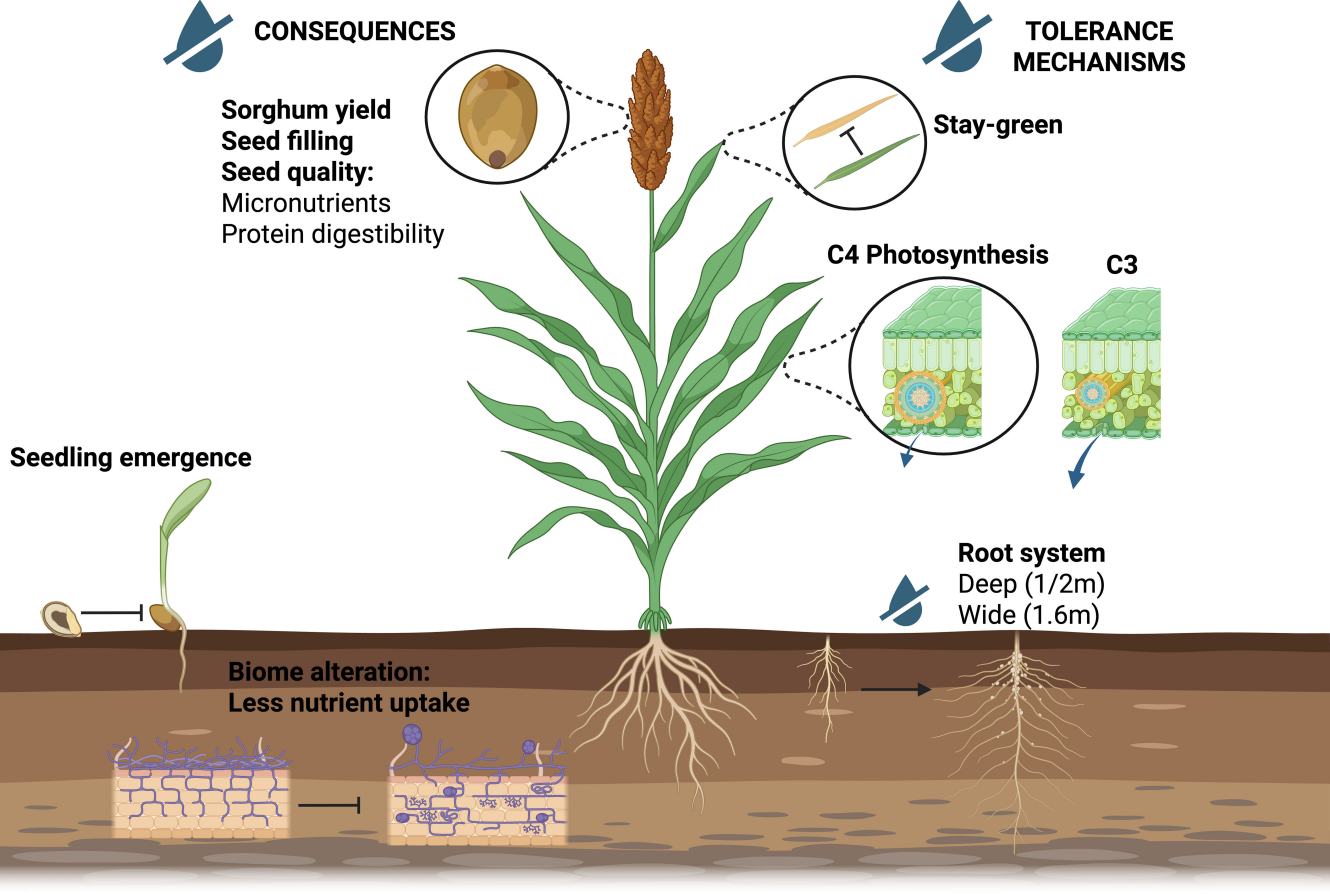
The impact of drought on Sorghum and its tolerance mechanisms. Drought stress affects Sorghum development at various stages, including seedling emergence, root establishment, and seed filling. However, Sorghum possesses several mechanisms to cope with this abiotic stress, such as deep and extensive root system, C4 photosynthesis to minimize water loss, and stay-green traits. Created in BioRender. Fontanet-Manzaneque, J. B. (2025) https://BioRender.com/91z4p60.

Sorghum is a key crop in water-limited environments. Its drought resilience is driven by a combination of morphological, physiological, and genetic adaptations that allow the plant to maintain productivity under water stress conditions (**Figure 2**). Physiological drought tolerance can be broadly defined as the plant’s ability to sustain photosynthetic carbon assimilation and regulate transpiration under water deficit conditions (Tardieu et al., 2018).

In the first physiological component, light capture and assimilation, Sorghum exhibits the retention of photosynthetically active leaves during periods of post-flowering drought, thereby extending the grain-filling period, a phenomenon known as the stay-green trait (Stg) (Borrell et al., 2000). This complex trait, involving different physiological processes, has been linked to four major quantitative trait loci (QTLs) known as *Stg1–4* which are responsible for half of the phenotypic variance observed in stay-green genotypes (Xu et al., 2000; Sanchez et al., 2002). Subsequent studies have revealed that each Stg individually reduces post-flowering drought-induced leaf senescence, and *Stg2* has been identified as the most prominent QTL, showing the greatest delay in leaf senescence, the highest green leaf area at maturity, and the lowest senescence rates among the individual QTLs (Harris et al., 2007). In experiments studying the interactions of these QTLs to determine the percentage of explained phenotypic variation, Stg2 alone accounted for 30% of the variation, and the Stg2+Stg3 combination explained nearly the 50% (49.8%), exceeding the sum of all individual effects (Subudhi et al., 2000; Xu et al., 2000).

In the second component, transpiration, the presence of each individual QTL (*Stg1-4*) in Sorghum cultivars is also characterized by a reduction in canopy and tillering at the anthesis stage, leading to a decrease in transpirational leaf area and better conservation of soil water prior to anthesis, for subsequent use during grain filling (Borrell et al., 2014). In this regard, it is noteworthy that the relationship between Stg traits and yield, although influenced by both environmental conditions and genetic background, exhibits a positive association in environments where yield is below 6 tons/ha. Consequently, the adoption of Sorghum hybrids with Stg could prove beneficial in enhancing yields across a wide range of environments, particularly since average yields worldwide are below 2.5 tons/ha (Jordan et al., 2012).

Additionally, Sorghum possesses the C4 photosynthetic pathway, which is crucial for its drought tolerance (**Figure 2**). Unlike many other plants that utilize the C3 photosynthesis pathway, in which CO_2_ is initially converted to a C3 compound, Sorghum rapidly produces C4 compounds using a specialized enzyme, phosphoenolpyruvate carboxylase, thereby reducing water loss through stomata (Pennisi, 2009). The CO_2_-concentrating mechanisms in C4 plants also avoids the oxygenase activity of RuBisCO, which is induced upon stomatal closure, thereby reducing photorespiration and improving carbon-use efficiency (Sage et al., 2012). This photosynthetic advantage, typical of plants adapted to harsh environments, relies on a specialized leaf anatomy known as Kranz anatomy. This anatomy, mainly defined as two distinct concentric layers of chlorenchyma cells, formed by a bundle sheath, surrounded by an outer layer of mesophyll cells, allows the segregation of the C4 synthesis and Calvin cycle in different cell types, having a CO_2_ concentration in the chlorenchyma cells (Lundgren et al., 2014).

Stomatal regulation is a central physiological mechanism enabling Sorghum to maintain productivity under drought conditions by balancing water conservation and carbon assimilation (Tari et al., 2013). Genotypic variability in stomatal behavior reflects distinct drought coping strategies: isohydric genotypes, such as ‘Gadambalia’, tend to close their stomata early to conserve water, though at the cost of reduced photosynthesis; whereas anisohydric types like ‘Tabat’ maintain stomatal opening and gas exchange even at lower water potentials (Tsuji et al., 2003).

Recent research has demonstrated that sorghum exhibits rapid stomatal kinetics, adjusting conductance within minutes of environmental changes. This dynamic response helps sustain photosynthesis and improves intrinsic water-use efficiency (WUE) under fluctuating conditions (Battle et al., 2024). Studies also associate faster stomatal closure, higher stomatal density, and narrower operational apertures with enhanced WUE, without compromising carbon assimilation (Al-Salman et al., 2023).

Considerable genetic variation among Sorghum genotypes has been reported in traits such as net carbon assimilation rate (A), transpiration rate (E), the A:E ratio, and WUE, under both well-watered and drought conditions. Increases in A:E and WUE have been linked to improved drought resilience, particularly during the pre-flowering stage (Balota et al., 2008). The genetic basis of stomatal conductance has also been explored, with associated QTLs identified (Lopez et al., 2017).

Beyond stomatal regulation, Sorghum also minimizes non-stomatal water loss through the deposition of epicuticular waxes on leaf surfaces. These hydrophobic layers form a barrier against cuticular transpiration, particularly under low relative humidity, to increase WUE by reducing the cuticular conductance to water vapor (Surwenshi et al., 2010).

Development and phenotypic analysis of wax-deficient mutants has facilitated the identification of key genetic components involved in cuticle and wax biosynthesis (Peterson et al., 1982; Jenks et al., 2000; Peters et al., 2009). These include QTL mapping, association studies, and map-based cloning (Burow et al., 2008, 2009; Awika et al., 2017; Punnuri et al., 2017; Uttam et al., 2017; Elango et al., 2020). More recently, spatial and developmental variation in wax composition across sorghum organs has been explored, alongside gene expression profiling of wax-related genes (Chemelewski et al., 2023).

In addition to its physiological advantages, the morphological feature of Sorghum that contributes to its drought tolerance is its root system (**Figure 2**). Sorghum roots can penetrate depths of 1 to 2 meters and efficiently extract water up to a lateral distance of 1.6 meters from the plant. This deep root system allows Sorghum plants to access moisture from deeper soil layers (Krupa et al., 2017). Experimental studies have demonstrated that Sorghum plants with deeper root systems exhibit higher yields and greater dry matter weight, which also influences the photosynthetic machinery (Chen et al., 2020). Root removal at 30 or 60 cm depths caused significant reductions in dry matter accumulation, photosynthesis and ultimately yield (Chen et al., 2020). In addition, several comparative studies with maize have further supported the direct correlation of deeper root systems with better drought adaptation. While maize exhibits higher above-ground dry weight in control conditions, Sweet sorghum showed a 27.2% increase in above-ground dry weight and a 200% increase in root dry weight under drought conditions in comparison with maize, highlighting the functional value of sorghum root biomass in drought tolerance (Schittenhelm and Schroetter, 2014).

Moreover, genetic studies have shown that QTLs for root dry weight (qRDW) and nodal root angle (qRA), which affects root density and distribution in the soil, are co-located with QTLs for stay-green traits and associated with better drought adaptation (Mace et al., 2012). This indicates that root angle and architecture play a role in influencing yield under drought stress conditions, even though there may not be a direct relationship with plant size (Singh et al., 2011).

QTL pyramiding has emerged as a strategic breeding approach in sorghum to enhance drought tolerance and productivity. This involves the combination of multiple beneficial QTLs into a single genotype using marker-assisted selection, though the process is labor-intensive and requires careful compatibility of gene activity (Kadam and Fakrudin, 2017; Nagaraja Reddy et al., 2013). When applied to traits like stay-green, pyramiding improves the potential expression of drought tolerance, as demonstrated by introgression lines containing *Stg3 Stg1 and Stg2*, which showed only a 10% yield reduction under water-stressed conditions compared to 18–23% of other Stg combinations, indicating enhanced tolerance (Kamal et al., 2018). Ultimately, only QTL combinations that demonstrate consistent expression across multi-environment trials are selected for varietal release, ensuring both yield stability and stress resilience (Gorthy et al., 2017; Kebede et al., 2001).

Summarizing the genetic linkage of the described traits, three main clusters can be identified: Stg1 and Stg2 QTLs on chromosome 3; Stg3 and the root dry weight QTL qRDW1_2 on chromosome 2; and Stg4, qRDW1_5 and the root angle QTL qRA1_5 on chromosome 5 (Xu et al., 2000; Mace et al., 2012; Deshpande et al., 2017).

## 3 Effects of drought stress on Sorghum

Despite being a drought-tolerant crop and thriving under low-input conditions, drought stress remains the most prevalent abiotic stressor faced by Sorghum in key production regions (**Figure 1**; Abreha et al., 2022). Drought stress impacts Sorghum development from the early stages, with seedling death during emergence and establishment being a common occurrence in drylands (**Figure 2**). Drought significantly hinders the germination rate in Sorghum (Smith et al., 1989). Subsequent studies have identified that the primary losses due to drought occur at various stages of early seedling development, including germination, emergence, seedling growth, and the lengths of coleoptiles, mesocotyls, radicles, and primary shoots (Bayu et al., 2005). Further investigations demonstrated the negative early effects of drought on germination rate and time, root and shoot length, dry matter, and seedling vigor (Queiroz et al., 2019).

Additionally, drought stress impacts Sorghum’s ability to uptake, mobilize and transport soil nutrients by altering the root-associated microbiome, specifically affecting symbiotic arbuscular mycorrhizal fungi (Symanczik et al., 2018; **Figure 2**).

Ultimately, drought is affecting its primary production trait, yield (Sarshad et al., 2021). However, the impact of water deficit on yield is highly dependent on growth stage and timing. Drought stress can reduce Sorghum yield by up to 36% when it occurs during the vegetative stage, and by as much as 55% during reproductive stages (Assefa et al., 2010). Furthermore, drought not only adversely affects Sorghum yield but also seed filling and grain quality (Sehgal et al., 2018; **Figure 2**). Total starch, amylose, and amylopectin content are reduced in grains exposed to drought during flowering (Bing et al., 2014). Water deficit also decreases protein digestibility and reduces micronutrient content, specifically zinc, manganese, and copper, in Sorghum grain nutrition (Impa et al., 2019).

Given the significant impact of drought stress on Sorghum, considerable attention must be directed towards developing tolerant cultivars and implementing efficient mitigation strategies in Sorghum production. Such strategies may include the use of early maturing varieties (Yahaya and Shimelis, 2022), optimized irrigation practices such as low irrigation at panicle initiation and termination at grain filling (Araya et al., 2018), marker-assisted selection and exploitation of stay-green traits (Mwamahonje et al., 2021), and genome editing approaches to develop improved drought-tolerant varieties.

## 4 Genetic and multi-omics resources available to improve abiotic stress resistance in sorghum

### 4.1 Sources of genetic variation for drought tolerance

While most cultivars and varieties of sorghum often comprise a very narrow genetic diversity, a valuable and underexploited source of drought tolerance is found in sorghum landraces and wild sorghum relatives unconstrained by domestication or stringent breeding (Ahmed et al., 2024, Sauer et al., 2024; Enyew et al., 2022; Ochieng et al., 2021; Nagesh Kumar et al., 2021). Wild and landrace sorghum germplasm represents a broad pool of new alleles for traits of drought resistance that can be applied to sorghum breeding.

Capturing and ensuring the conservation of such diversity has been the objective of many repositories such as the USDA Agriculture Research Service National Plant Germplasm System, currently holding over 7200 accessions of Ethiopian sorghum lines (Cuevas et al., 2017). Likewise, utilizing chemical mutagenesis techniques such as Ethyl Methane Sulfonate (EMS) mutagenesis and Targeting Induced Local Lesion In Genomes (TILLING) is a proven approach for producing new sorghum germplasm (Jiao et al., 2016; Blomstedt et al., 2012; Xin et al., 2009; Mason et al., 2024; Xin et al., 2008; Kalpande et al., 2022), which have already enabled the basic study of loci implicated in drought response and tolerance mechanisms in Sorghum (Fontanet‐Manzaneque et al., 2024b; Harris-Shultz et al., 2019).

### 4.2 Genomic resources and advancements in pangenomics

Sorghum molecular research was greatly boosted by the publication of the first reference genome (BTx623) in 2009 by Paterson et al., 2009 and further improved in McCormick et al. (2018), who already envisioned Sorghum, with its relatively small genome of around 800 Mb and 34,211 bona fide protein-coding genes, as a potential model species for C4 grasses. Molecular breeding approaches of Sorghum heavily rely on the BTx623, Tx430 (Deschamps et al., 2018) and Rio (Cooper et al., 2019) accession assemblies, with the BTx623 accession being the most used as a parent for grain hybrid generation.

Owing to the technical advancements, whole-genome sequencing in population and quantitative genomics studies has been useful for characterizing the variability of agronomic traits in sorghum (Faye et al., 2019; Morris et al., 2013). Resources like the West African sorghum association panel (WASAP), consisting of sorghum landraces and breeding lines from West Africa, allow the study of agronomical traits of interest (Faye et al., 2021a) with diverse accessions. WASAP has been applied in GWAS-based studies for identifying novel drought-response associated loci (Faye et al., 2021b; Maina et al., 2022), and pleiotropic loci were found to be associated with various drought tolerance traits in pre- and post-flowering drought scenarios. Furthermore, genome–environment association studies, which are relevant for identifying genotype-by-environment interactions and predicting phenotypic variation, have also been reported for drought and other climatic adaptation traits (Girma et al., 2020; Menamo et al., 2021; Lasky et al., 2015).

However, understanding drought adaptation needs the added perspective of studies tackling structural variation and gene presence/absence patterns in a broad number of genotypes beyond a single reference genome, an area where pangenomics is particularly valuable. Pangenomes are a powerful tool for genetic variability characterization, as has been evidenced for species such as wheat (Jiao et al., 2025), rice (Guo et al., 2025), and other agronomically important species.

The recently published sorghum pangenome (Tao et al., 2021) constructed with 13 cultivated genomes and 5 wild inter-fertile relatives genomes reveals a high number of dispensable genes (this is, genes unique between genome accessions), among which many Copy Number Variants and Presence/Absence Variants are enriched in abiotic and biotic stress Gene Ontology processes, highlighting again this underexploited source of genetic diversity. In the case of drought, Ruperao et al. (2021) mapped available transcriptomics data from contrasting resistant vs. susceptible genotypes to a sorghum pangenome and found 1,788 genes deregulated by drought, out of which 79 were newly identified with the pangenome assembly. A new study by Cole et al. (2025) employed a pangenome assembly of three sorghum accessions, BTx642 (a Stg genotype), Tx430, and BTx623 (senescent genotypes), to evaluate consistent drought-responsive gene expression across Tx430 and BTx623 over three consecutive years, thereby minimizing artifacts associated with using a single reference genome.

### 4.3 Omic profile of Sorghum’s drought adaptation

The development of integrated omics databases, such as those compiled by Liu et al. (2024), represents a valuable resource for the genetic improvement of Sorghum. Genomics, transcriptomics, proteomics, metabolomics and phenomics studies have evaluated Sorghum’s responses to drought. As a result, many of the specific genes and pathways involved in its drought adaptation mechanisms have been identified (**Table 1**).

**Table 1:** Summary of currently available genetic tools in Sorghum.

Protein name	Functional characterization	Identification	Gene Ids	Reference
LATE EMBRYOGENESIS ABUNDANT	Protecting cells from abiotic stress	Transcriptomics	*Sb01g046490, Sb09g027110, Sb07g015410, Sb04g017790, Sb03g032380, Sb01g021320, Sb03g001170*	Johnson et al. (2014); Abdel-Ghany et al. (2020); Zhang et al. (2019)
DELTA 1-PYRROLINE-5-CARBOXYLATE SYNTHASE 2	Proline metabolism	Transcriptomics	*Sb03g039820*	Johnson et al. (2014, 2015); Varoquax et al. (2019); Cole et al. (2025)
HIGH-AFFINITY K+ TRANSPORTER 1	Sodium ion transmembrane transporter	Transcriptomics	*Sb06g027900*	Johnson et al. (2014)
EXPANSINS	Cell wall loosening	Transcriptomics	*Sb06g026480, Sb10g024380, Sb03g038290, Sb04g009990, Sb07g001540, Sb03g005140, Sb04g032830*	Johnson et al. (2014); Fracasso et al. (2016); Zhang et al. (2019)
GLUTATHIONE TRANSFERASES	Antioxidant	Transcriptomics	*Sb03g031780*	Abdel-Ghany et al. (2020); Varoquax et al. (2019)
DEHYDRATION-RESPONSIVE ELEMENT-BINDING	Enhancing abiotic stress tolerance	Transcriptomics	*Sb02g030310, Sb02g030300*	Abdel-Ghany et al. (2020)
HEAT SHOCK PROTEINS	Proteostasis and buffer stresses	Transcriptomics	*Sb10g027230, Sb06g000660, Sb03g003530, Sb03g034390, Sb03g006920, Sb01g041180, Sb01g025610*	Abdel-Ghany et al. (2020); Zhang et al. (2019)
DEHYDRATION-RESPONSIVE ELEMENT-BINDING 2	Enhancing abiotic stress tolerance	Heterologous expression	*Sb03g004980*	Bihani et al. (2011); Izadi-Darbandi et al. (2023)
NAC1	Enhancing drought tolerance	Heterologous expression	*Sb01g003710*	Lu et al. (2013)
WRKY30	Influence root architecture, proline content, and ROS scavenging	Heterologous expression	*Sb10g004000*	Yang et al. (2020)
WIN1 LIKE 1	Wax and cutin accumulation	Heterologous expression	*Sb04g006970*	Bao et al. (2017)
ERECTA2	Increased drought tolerance	Heterologous expression	*Sb04g034820*	Li et al. (2019)
STRESS-ASSOCIATED PROTEIN 14	Enhance tolerance to salt stress and oxidative damage	Heterologous expression	*Sb01g005640*	Wang et al. (2013)
ALKALINE TOLERANCE 1	Regulate flux of hydrogen peroxide by regulating aquaporins	Genetic modification of Sorghum	*Sb01g032830*	Zhang et al. (2023)
NAC19	Enhanced drought tolerance and increased ROS scavenging	Genetic modification of Sorghum	*Sb05g005450*	Jin et al. (2023)
MYC2	Enhanced drought tolerance and increased ROS scavenging	Genetic modification of Sorghum	*Sb01g028230*	Wang et al. (2024)
PIN FORMED 1, 2 and 4	Stay-green; Auxin efflux carrier	Genetic modification of Sorghum	*Sb02g029210, Sb03g029320, Sb03g037350*	Borrell et al. (2022)

### 4.4 Transcriptomics insights on drought responsive gene expression

Transcriptome profiling of Sorghum under drought stress has revealed key adaptive mechanisms. Among the most responsive genes, LATE EMBRYOGENESIS ABUNDANT (LEA) proteins, the sodium transporter HIGH-AFFINITY K+ TRANSPORTER 1 (HKT1), and the DELTA 1-PYRROLINE-5-CARBOXYLATE SYNTHASE 2 (P5CS2), involved in proline metabolism, were notably upregulated under drought conditions (Johnson et al., 2014; Abdel-Ghany et al, 2020; Zhang et al., 2019; Varoquaux et al., 2019). Later studies have consistently highlighted the importance of P5CS2 in drought adaptation. For example, P5CS2 is significantly upregulated in the Stg genotype B35 compared to the senescent genotype R16, and it maps within the Stg1 QTL (Johnson et al., 2015). However, a pangenome-based transcriptomic analysis expanding on the dataset of Varoquaux et al. (2019) identified P5CS2 as a core drought-responsive gene, being upregulated in both a Stg and a senescent genotype (Cole et al., 2025). This suggests that P5CS2-mediated proline accumulation may represent a general drought response mechanism rather than a stay-green–specific adaptation. Deregulation of glutathione transferases is also a common mechanism across Sorghum varieties in response to water deficit, suggesting a conserved drought response. Indeed, lower constitutive expression of glutathione transferase genes could explain the sensitivity upon water stress of drought-sensitive varieties (Abdel-Ghany et al., 2020; Fracasso et al., 2016).

Additionally, upregulation of expansins and HEAT SHOCK PROTEINS (HSPs) is a conserved mechanism of Sorghum to drought stress and in combination with heat stress (Johnson et al., 2014; Fracasso et al., 2016; Zhang et al., 2019). However, specific changes observed in drought-resistant varieties include upregulation of transcription factors such as *DEHYDRATION-RESPONSIVE ELEMENT-BINDING PROTEINS* (*DREBs*) and specific HSPs (Abdel-Ghany et al., 2020).

At transcript level, Sorghum possesses conserved responses involving deregulation of LEAs, HSPs, P5CS2, expansins and glutathione transferases (e.g., GST29), while stress-resilient varieties further exhibit elevated expression of DREBs and specific HSPs.

### 4.5 Proteomic signatures of drought adaptation in Sorghum

Compared to transcriptomics, proteomics offers a more accurate picture of the cellular state, as proteins are the actual effectors of biological activity. Proteomics data reveal post-transcriptional regulatory patterns that transcript data alone cannot provide (Naaz et al., 2024; Satrio et al., 2024).

In response to drought, Ngara et al., 2018 found that sorbitol-induced osmotic stress in Sorghum triggered increased abundance of proteins targeted to the secretory pathway, including expansins, redox proteins, proteases, and glycosyl hydrolases. In a comparative proteomic study, Goche et al., 2020 reinforced the relevance of fast stomatal closure as a key trait for drought tolerance. Other signature mechanisms seen in tolerant sorghum genotypes include the accumulation of the osmoprotectant glycine betaine and root architectural modifications that increase the root-to-shoot ratio, thereby improving water uptake efficiency.

Recently, a comprehensive quantitative proteomics dataset of two contrasting sorghum drought genotypes revealed distinct patterns of protein accumulation in leaves and roots under drought, heat, and combined stress conditions (Ali et al., 2025), providing a valuable resource to identify protein markers associated with drought tolerance and support Sorghum breeding programs.

### 4.6 Metabolomic profile in response drought in Sorghum

Metabolomics allows for direct profiling of the end products of drought stress response. Baker et al. (2023) investigated post-flowering drought responses of the reference genotypes BTx642 and RTx430, and observed distinct, genotype-specific accumulation of osmolytes. Metabolites such as galactinol, α-ketoglutarate, and aspartate positively correlate with stomatal conductance and decline as drought induces stomatal closure. In contrast, fumarate and maleate accumulate during drought and correlate inversely with stomatal opening, illustrating metabolic adaptation to water stress (Baker et al., 2023). Another untargeted metabolomics study explored how four genotypes (agriculturally low- or high-performing) modulate root metabolites to shape their rhizosphere microbiome under drought: the metabolome of low-performing genotypes was enriched in flavonoids, while high-performing genotypes showed greater modulation of other compounds, including pipecolinic acid, 13-(S)-hydroxyoctadecatrienoic acid (an oxylipin), and albiflorin (a terpenoid) (García et al., 2025). These findings suggest distinct metabolic strategies in root-microbiome interactions during drought. Further studies could aim to define consistent metabolic signatures linked to drought tolerance in sorghum.

### 4.7 Phenomics

Phenomics, which analyzes the set of observable traits in an organism, has become an essential tool for capturing genetic variation under field-relevant environmental conditions. As genomics and genotyping have become more accessible, phenotyping remains the main bottleneck in linking genetic variation to traits, due to the laborious nature and high costs of measuring traits at large scale and high throughput. Advances in imaging, sensors, and data analysis have made phenomics a valuable tool in sorghum, both for evaluating agronomic and drought tolerance traits (Bao and Tang, 2016; Salas Fernandez et al., 2017; Watanabe et al., 2017; Jadhav et al., 2024). Spindel et al. (2018) carried out a GWAS in sorghum by employing aerial drone imagery to capture phenotypic data. By analyzing patterns of historical recombination, the study identified specific genetic variants linked to key traits such as drought tolerance and biomass yield.

### 4.8 Integrative multi-omics for developing drought tolerant cultivars

The integration of omics approaches, while inherently challenging at the technical and analytical level, offers a more holistic understanding of complex biological processes, especially those as complex as drought response. Instead of isolated molecular markers or singular responses, multi-omics approaches identify regulatory networks and molecular interactions that collectively contribute to stress resilience in sorghum (Mukherjee et al., 2024; Seth et al., 2025; Ren et al., 2022). In this context, Yue et al. (2025) combined metabolomic and transcriptomic analyses to investigate drought responses and reaffirmed the involvement of the flavonoid biosynthetic pathway. Their results revealed that certain flavonoid biosynthesis genes correlated positively with metabolite levels, while other showed negative correlations, indicating a precise regulation under stress.

Further supporting the role of flavonoids in sorghum’s drought tolerance, Fontanet-Manzaneque et al. (2024b) employed transcriptomics, cistromics and metabolomics to demonstrate that SbBRI1 brassinosteroid receptor signaling is attenuated under drought, facilitating activation of the flavonoid pathway via BES1 transcription factor.

These integrative omics not only reveal mechanisms of stress adaptation but also directly contribute to and streamline the development of new cultivars, as they enable the identification of key genes, pathways, and observable traits associated with stress tolerance much more rapidly. Coupling this multilayered data with modern breeding techniques, such as genomic selection and marker-assisted selection (MAS), can significantly accelerate and improve the development of stress-tolerant cultivars (Hao et al., 2021; Liaqat et al., 2024).

## 5 Functional validation and genetic engineering of drought-responsive genes

More targeted approaches have addressed the functional validation of specific genes involved in drought tolerance mechanisms in Sorghum. However, due to the technical challenges associated with stable sorghum transformation, many genes have been validated using heterologous systems such as *Arabidopsis thaliana*, rice, or maize, species that are more amenable to genetic manipulation. While informative, these systems do not always replicate the full physiological context of Sorghum, and functional results may not fully translate across species.

Transcription factors such as SbDREB2, SbNAC1, and SbWRKY30 have been functionally validated in rice, maize, and *Arabidopsis*, where their overexpression consistently enhanced drought tolerance (Bihani et al., 2011; Lu et al., 2013; Yang et al., 2020; Izadi-Darbandi et al., 2023). Other engineering efforts include SbWINL1, a wax-inducing transcription factor that increased drought tolerance in Arabidopsis through the induction of wax and cutin biosynthesis (Bao et al., 2017); SbER2, a receptor-like kinase whose expression in maize and Arabidopsis improved water-use efficiency and photosynthetic performance under drought (Li et al., 2019); and SbSAP14, a STRESS-ASSOCIATED PROTEIN that mitigated salt-induced oxidative stress and delayed leaf senescence when expressed in rice (Wang et al., 2013).

Despite these advances in heterologous systems, only a few laboratories have successfully transformed sorghum to directly investigate gene function in planta. Notably, a Genome-Wide Association Study identified ALKALINE TOLERANCE 1 (AT1), which encodes an atypical G protein γ subunit. In Sorghum, overexpression of AT1 reduced alkaline tolerance, whereas knockout lines displayed enhanced stress resistance through increased regulation of H_2_O_2_ efflux via aquaporin phosphorylation (Zhang et al., 2023). This study highlights the mechanistic insights that can be obtained from direct genetic manipulation in Sorghum itself.

Additional examples of successful sorghum transformation include SbNAC9, whose overexpression conferred drought tolerance through improved detoxification of reactive oxygen species (ROS) (Jin et al., 2023), and SbMYC2, a bHLH transcription factor that reduced ROS accumulation under drought conditions. In contrast, silencing SbMYC2 compromised stress tolerance in sorghum seedlings (Wang et al., 2024).

Furthermore, three PIN-FORMED auxin transporters, SbPIN1, SbPIN2, and SbPIN4, linked to the Stg1–3 QTLs, were introduced into a non-stay-green sorghum cultivar. Their expression led to improved canopy development, enhanced root architecture, and increased panicle growth. Notably, these lines also exhibited reduced leaf area, which may contribute to lower transpiration rates under drought conditions (Borrell et al., 2022).

## 6 Sorghum transformation

Despite the identification of specific genes involved in drought-tolerant mechanisms, there is still limited progress in genetic engineering of sorghum varieties. Sorghum is still considered a crop recalcitrant to genetic transformation (Visarada and Kishore, 2015; Miller et al., 2023). Optimizing transformation efficiency is crucial for the development of Sorghum cultivars better adapted to the current climate change context.

### 6.1 Sorghum transformation limitations

Sorghum is considered the most recalcitrant crop among cereals for tissue culture due to the accumulation of phenolic compounds and a high degree of genotype dependence (Azhakanandam and Zhang, 2015).

Phenolic compound accumulation remains a primary bottleneck in Sorghum transformation. Several factors induce the accumulation of phenolic pigments in Sorghum *in vitro* cultures. *Agrobacterium tumefaciens* (Agrobacterium) infection and phosphinothricin selection trigger stress responses that lead Sorghum cells to release toxic levels of phenolics, compromising cell viability (Tadesse et al., 2003; Zhao et al., 2000).

Various strategies have been adopted to minimize phenolic compound accumulation such as performing short subculture intervals and adding 1% polyvinylpolypyrrolidone (Cai et al., 1987; Zhao et al., 2000); changing the selection system to mannose (Gao et al., 2005); changing phosphinothricin selection to geneticin (Tadesse et al., 2003); using activated charcoal to reduce black pigment production (Nguyen et al., 2007); and cold pre-treatment of immature seeds positively affecting both explant survival and callus formation while reducing phenolic compounds, likely due to the inhibition of key enzymes involved in phenolic compound synthesis (Dicko et al., 2006). Supplementing the media with L-asparagine (6.7 mM), L-proline (17.4 mM), and different concentrations of NO_3_^-^, NH_4_^+^, and PO_4_^-^ resulted in increased induction and growth of Sorghum friable embryogenic callus without medium pigmentation (Elkonin and Pakhomova, 2000). Combining this supplemented media with geneticin selection further mitigated the compounding effect of phenolic secretion (Howe et al., 2006). These phenolic mitigation strategies are now widely integrated into modern protocols and are summarized in **Table 2**, alongside transformation efficiencies.

**Table 2:** Summary of the progress in Sorghum transformation.

Reference	Genotype	Method	Morphogenic regulators	Ternary vector	Optimization	Efficiency
Casas et al., 1993	P898012	Bombardment			Immature embryo	0,3% (a)
Zhao et al., 2000	P898012	Agrobacterium		Super-binary	Adding PVPP, short subcultures and source of the embryo	10% (b)
Tadesse et al., 2003	Ethiopian accession 214856	Bombardment			Bombardment parameters and geneticin selection	1,08% (a)
Gao et al., 2005	Pioneer 8505	Agrobacterium			Changing selection to mannose	2,88% (a)
	C401					3,3% (a)
Nguyen et al., 2007	Sensako 85/1191	Agrobacterium			Activated charcoal	5% (b)
Gurel et al., 2009	P898012	Agrobacterium			Heat treatment of embryos	7% (a)
Liu and Godwin, 2012	Tx430	Bombardment			DNA delivery conditions	20,7% (b)
Wu et al., 2014	Tx430	Agrobacterium		Super-binary	Optimized from Zhao et al., 2000	33.2% (b)
Lowe et al., 2016	Tx430	Agrobacterium	BBM and WUS2	Super-binary	Use of morphogenic regulators	18% (b)
Che et al., 2018	Tx430	Agrobacterium		Ternary vector	Ternary vector	29%(b)
	Malisor 84-7					9%(b)
	Tegemeo					9,3%(b)
	Macia					1%(b)
Che et al., 2022	Tx430	Agrobacterium	BBM and WUS2	Ternary vector	Combination of ternary vector and morphogenic regulators	69,7%(b)
	Malisor 84-7					21,7%(b)
	Tegemeo					17,1%(b)
	Macia					20%(b)
Li et al., 2024	PI655975	Agrobacterium	GRF4-GIF1	Ternary vector	GRF4-GIF1 morphogenic regulators	39,68% (a)
Fontanet-Manzaneque et al., 2024a	Tx430	Agrobacterium	BBM and WUS2	Ternary vector	Bacterial density, co-cultivation time, temperature	164,8% (b)

Transformation efficiencies: (a) Number of embryo with regenerated shoots per 100 embryos infected. (b) Number of regenerated shoots recovered per 100 embryos infected.

Genotype dependence poses an equally critical limitation in Sorghum transformation. Sorghum callus induction and regeneration have also been quite limited by genotype. Even with established transformation methods, many elite and agronomically important varieties remain poorly responsive (Botella, 2019; Kaeppler and Pedersen, 1997; Hagio, 2002).

However, the implementation of new methodologies to ameliorate phenolic toxicity and increase regeneration frequencies has enabled the transformation of previously un-transformable elite varieties, such as Ramada, Malisor 84-7, Tegemeo and Macia (Raghuwanshi and Birch, 2010; Liu et al., 2015; Omer et al., 2018; Nelson-Vasilchik et al., 2018; Che et al., 2018; Flinn et al., 2020).

Overcoming genotype recalcitrance and phenolic accumulation is thus central to unlocking the full potential of Sorghum as a next-generation model system and as a resilient cereal crop for climate-adaptive agriculture.

### 6.2 Sorghum transformation progress

#### 6.2.1 Microprojectile bombardment

The first successful genetic transformation of Sorghum was achieved using microprojectile bombardment using immature embryos as explants (Casas et al., 1993). Although various explants such as immature inflorescences, shoot tips, mature embryos, embryogenic calli, and leaf whorls have been used for Sorghum transformation, immature embryos remain the predominant target (Brettell et al., 1980; Tadesse et al., 2003; Silva et al., 2020).

The first report of sorghum transformation was achieved with microprojectile bombardment reaching 0.3% transformation efficiency (Casas et al., 1993) Optimization of bombardment parameters, including acceleration pressure, distance to the target, aperture of the helium inlet valve, gap width, and microprojectile travel distance, improved efficiency to 1.08% (Able et al., 2001; Tadesse et al., 2003). Further refinements in culture media ingredients, along with optimized DNA delivery conditions increased efficiency to 20.7% (Liu and Godwin, 2012).

#### 6.2.2 Agrobacterium-mediated transformation

Simultaneously, Agrobacterium-mediated Sorghum transformation was optimized through adjustments in infection, co-cultivation, and selection conditions. This included using different selecting agents, applying various treatments like cold or heat pretreatment to promote callus induction, and reduce phenolic compounds (Gao et al., 2005; Nguyen et al., 2007; Gurel et al., 2009). Despite these efforts, Agrobacterium-mediated transformation efficiency remained lower than particle bombardment until Wu et al. (2014) achieved a 33% efficiency using super binary vectors. Super binary vectors, which enhance the virulence of Agrobacterium, were first utilized in plant transformation with *Chenopodium quinoa* (Komari, 1990) and are binary vectors (small T-DNA carrying plasmid) including additional virulence genes (virB, virC, and virG) (Komari et al., 2006).

Since then, the adoption of Agrobacterium has become the predominant transformation protocol [Anand et al. (2018); Che et al. (2018); Hoerster et al. (2020); Che et al. (2022); Johnson et al. (2023); Wang et al. (2023); Li et al. (2024), and Fontanet-Manzaneque et al. (2024a)].

#### 6.2.3 Implementation of morphogenic regulators

The implementation of morphogenic regulators was a significant breakthrough in monocot transformation substantially increasing the transformation efficiencies of cereals, including Sorghum, maize, rice, and sugarcane (Lowe et al., 2016). This approach leveraged morphogenic transcription factors like maize BABY BOOM (BBM) and WUSCHEL2 (WUS), which promote the transition from vegetative to embryonic growth (Boutilier et al., 2002; Zuo et al., 2002), and increased Sorghum transformation frequencies from 1.9% to 18.3% with morphogenic genes present in the T-DNA of a super binary vector. However, to regenerate plants, it was necessary to remove BBM and WUS2 expression cassettes due to their pleiotropic effects. This was achieved using a drought-inducible maize promoter (p*RAB17*) to drive *CRE* recombinase gene expression, which excised the morphogenic regulators, flanked by loxP sites, upon desiccation (Lowe et al., 2016).

#### 6.2.4 Implementation of ternary vectors

The development of a ternary vector system further improved transformation efficiencies. Traditional super binary vectors (e.g., pSB1) have limitations due to their large size and the need for a co-integration step. To address these limitations, new “pVIR” vectors were created, featuring small size, enhanced stability, improved bacterial selectable markers, and an optimal set of amended *vir* genes (operons virC, virD, and virE) (Anand et al., 2018; Lowe et al., 2018). This introduction of the pVIR plasmid *in trans* with T-DNA binary vector in the same Agrobacterium strain enabled the development of a rapid maize transformation system (Lowe et al., 2018).

Che et al. (2018) combined the ternary vector system with media optimization techniques described by Wu et al. (2014), achieving high transformation efficiencies in recalcitrant African varieties, including Macia, Malisor 84-7, and Tegemeo. The combination of the ternary vector system, media optimization, and morphogenic regulators (*ZmWUS2* and *ZmBBM*) led to the highest reported Sorghum transformation efficiency of 69.7% (number of regenerated shoots recovered per 100 embryos infected) (Che et al., 2022). Additionally, the use of alternative morphogenic regulators like GRF4-GIF1 achieved transformation efficiencies of 39.68% (number of embryo with regenerated shoots per 100 embryos infected) (Li et al., 2024), demonstrating the versatility and effectiveness of these advanced transformation methodologies. Finally, Fontanet-Manzaneque et al. (2024a) combined the ternary vector pVS1-VIR2 with ZmBBM and ZmWUS2, and optimized the bacterial density, temperature and co-cultivation time leading to a 164.8% transformation efficiency (number of regenerated shoots recovered per 100 embryos infected), a 2.36-fold increase compared to Che et al., 2022.

**Figure 3:**
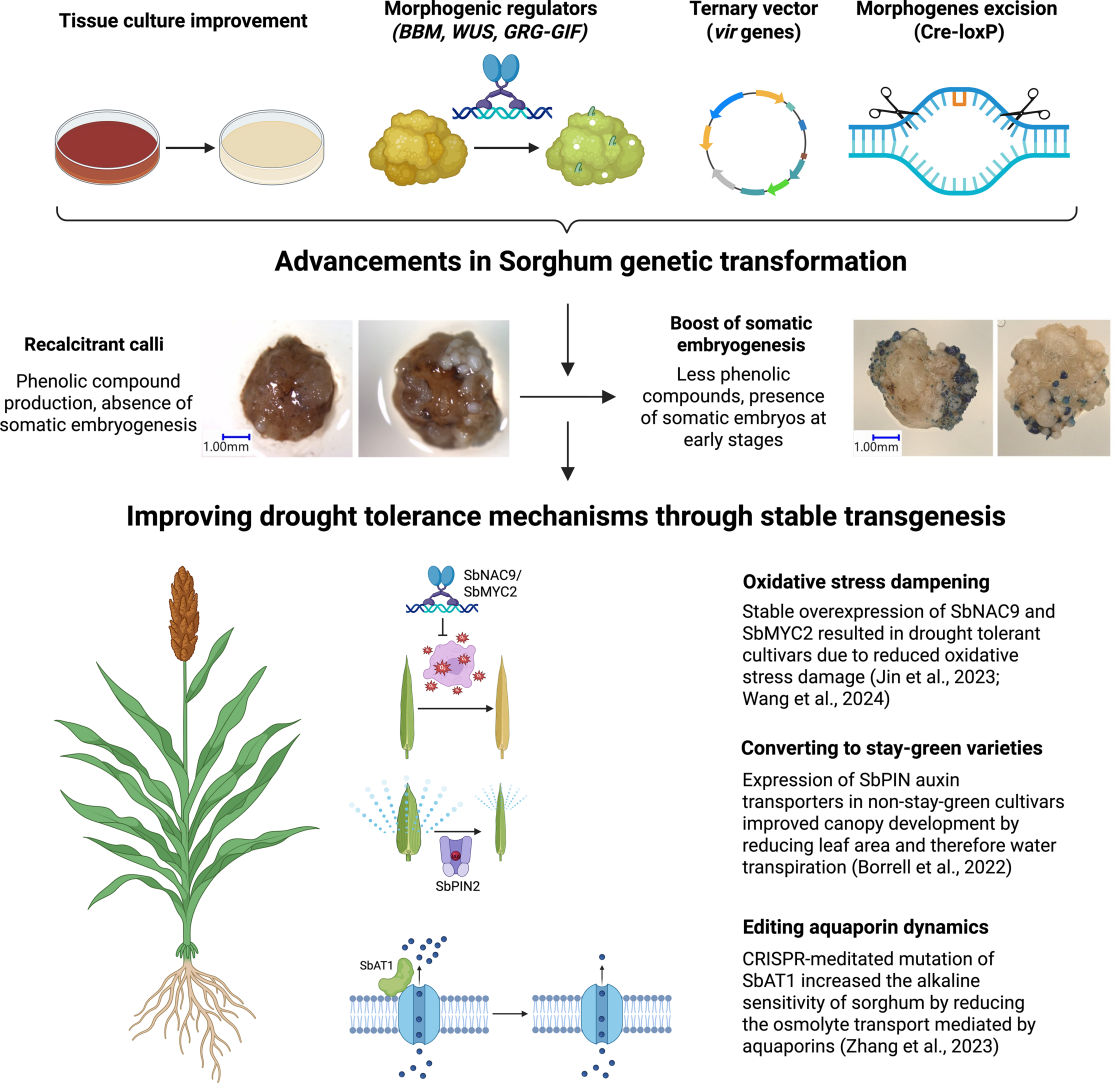
Advancements in Sorghum genetic transformation enable improvement of drought tolerance by stable transgenesis. The refinement of sorghum tissue culture and the development of new technologies including the use of morphogenic regulators, ternary vector system and morphogene excision have allowed the induction of somatic embryogenesis and overcome the sorghum calli recalcitrance. This paves the way to exploit the drought tolerance mechanisms of sorghum by stable transgenesis. Already existing examples reinforce the use of this technology to speed up the generation of crops better adapted to climate change Created in BioRender. Fontanet-Manzaneque, J. B. (2025) https://BioRender.com/xke8k9l.

The advancements in Sorghum genetic transformation have significantly progressed from the initial use of microprojectile bombardment to more sophisticated techniques involving Agrobacterium-mediated methods. The development and utilization of ternary vector systems, alongside media optimization and innovative morphogenic regulators, have culminated in unprecedented transformation efficiencies, even in recalcitrant Sorghum varieties. These methodological advancements not only enhance the feasibility of genetic transformation in Sorghum but also open new avenues for developing drought-resistant and climate-resilient Sorghum cultivars (**Figure 3**).

## 7 Closing remarks and future prospects

Plant biology has long relied on model organisms such as Arabidopsis to dissect fundamental biological processes. These model species offer distinct experimental advantages, including short life cycles, compact and well-annotated genomes, high transformation and gene editing efficiencies, and simple cultivation requirements. However, their limited agronomic relevance necessitates substantial translational effort to transfer basic findings into field applications. In contrast, crop research targets agriculturally important species (such as wheat, rice, and maize) to generate immediate impact, but progress is often hampered by longer generation times, complex polyploid genomes, and recalcitrance to transformation. Ultimately, both research paradigms face a major bottleneck when translating knowledge from the laboratory to the field.

**Figure 4:**
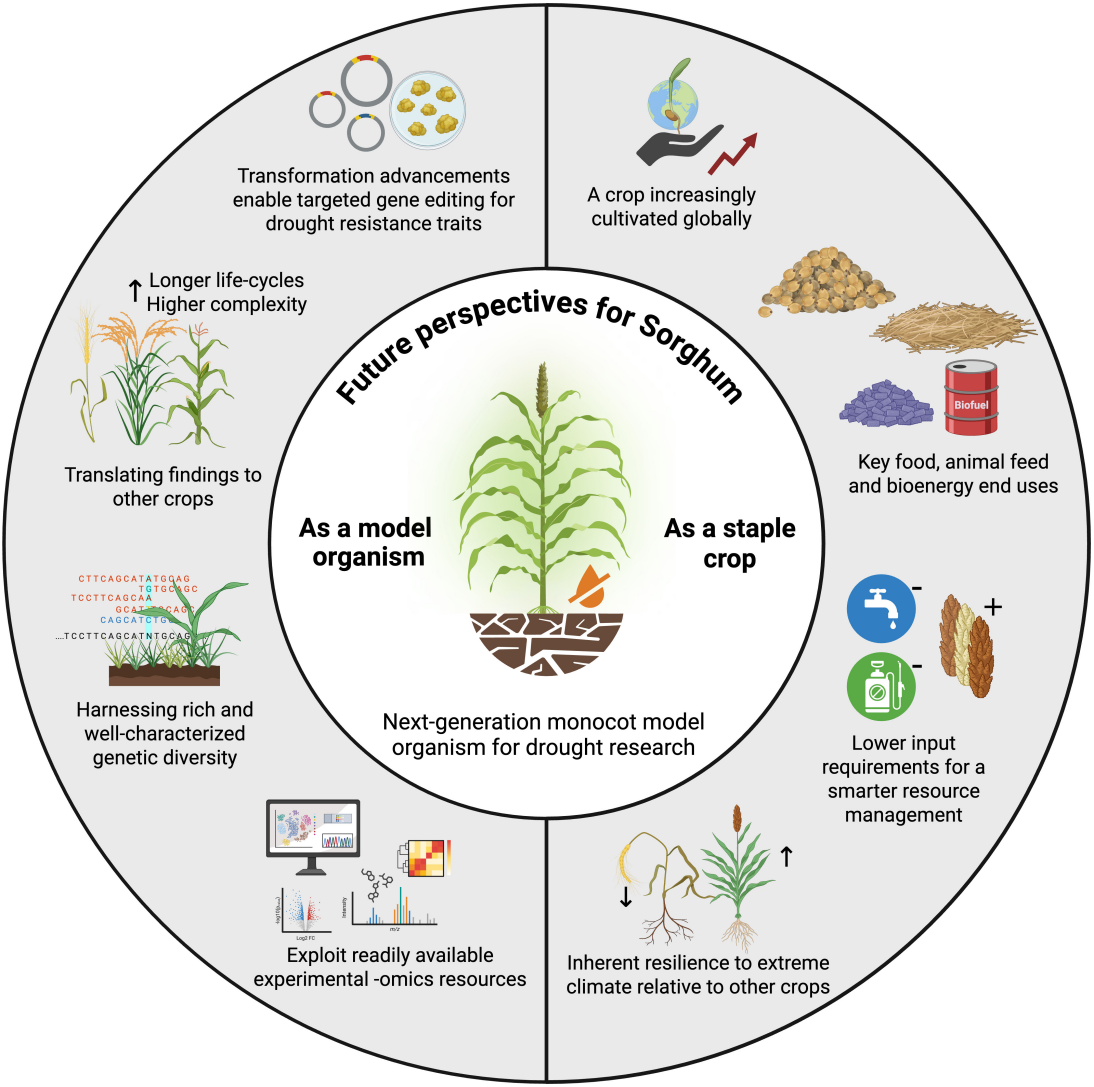
Future perspectives for Sorghum as model organism for the study of drought in monocots. Encompassing characteristics of a model plant and a staple crop, sorghum holds the potential of accelerating the development of climate-smart (particularly drought resistant) crops. Created in BioRender. Caño-Delgado, A. I. (2025) https://BioRender.com/496nmk2.

Sorghum combines several key features of model organisms—such as a small diploid genome, high genetic diversity, and growing omics resources—with the practical strengths of a staple crop: global cultivation, inherent drought tolerance, low input requirements, and multiple end-uses spanning food, feed, and bioenergy. These attributes make sorghum a promising system for both mechanistic studies of abiotic stress in monocots and direct crop improvement (**Figure 4**). However, important challenges remain before sorghum can be established as a model crop with the robustness and legacy of Arabidopsis.

First, spatial resolution is lacking. While recent transcriptomic studies have attempted to address this by using Laser Capture Microdissection to profile tissue-type-specific expression in sorghum secondary cell wall development (Fu et al., 2024), the field still lacks tissue-specific markers and single-cell resolution tools. Techniques such as single-cell and single-nucleus RNA-seq, already well-established in Arabidopsis and maize (Tenorio-Berrío et al., 2022; Doll et al., 2025), remain largely underdeveloped in Sorghum.

In addition, although many transcriptomic studies have characterized gene expression responses to drought (Johnson et al., 2014; Abdel-Ghany et al., 2020; Zhang et al., 2019; Varoquaux et al., 2019), very few have explored the cistrome or epigenomic landscape, which are crucial to understanding where transcription factors bind across the genome and how chromatin modifications, such as DNA methylation or histone acetylation, regulate gene expression. Recent efforts, such as the study by Zhou et al. (2021), have begun to address this gap using transposase-accessible chromatin sequencing (ATAC-seq) and bisulfite sequencing to detect epigenetic marks. However, more work is needed to map chromatin accessibility and regulatory regions across different tissues and environmental conditions.

Finally, despite Sorghum’s reputation as a drought-tolerant crop, its water footprint remains suboptimal compared to major cereals. For instance, producing one ton of sorghum grain requires 5,695 m³ of water, whereas maize and wheat require only 2,522 m³/ton and 2,474 m³/ton, respectively (Mali et al., 2021). This highlights an urgent need to improve sorghum yield and resource use efficiency through advanced breeding and biotechnological tools, such as CRISPR-Cas9 and the recent breakthroughs in stable genetic transformation, which historically represented one of the biggest bottlenecks in Sorghum research.

In conclusion, while several technical and biological challenges remain, Sorghum stands out not only as a resilient cereal crop but also as a forward-looking model system to bridge basic and translational plant science. Its increasing amenability to genomic tools, transformation protocols, and integrative multi-omics approaches positions it as a strategic platform for accelerating drought tolerance research and driving innovation in cereal crop improvement under climate stress.
